# Linking Vital Rates of Landbirds on a Tropical Island to Rainfall and Vegetation Greenness

**DOI:** 10.1371/journal.pone.0148570

**Published:** 2016-02-10

**Authors:** James F. Saracco, Paul Radley, Peter Pyle, Erin Rowan, Ron Taylor, Lauren Helton

**Affiliations:** 1 The Institute for Bird Populations, P.O. Box 1346, Point Reyes Station, CA, 94956–1346, United States of America; 2 Commonwealth of the Northern Mariana Islands, Division of Fish and Wildlife, Department of Lands and Natural Resources, P. O. Box 10007, Saipan, MP, 96950, United States of America; Cary Institute of Ecosystem Studies, UNITED STATES

## Abstract

Remote tropical oceanic islands are of high conservation priority, and they are exemplified by range-restricted species with small global populations. Spatial and temporal patterns in rainfall and plant productivity may be important in driving dynamics of these species. Yet, little is known about environmental influences on population dynamics for most islands and species. Here we leveraged avian capture-recapture, rainfall, and remote-sensed habitat data (enhanced vegetation index [EVI]) to assess relationships between rainfall, vegetation greenness, and demographic rates (productivity, adult apparent survival) of three native bird species on Saipan, Northern Mariana Islands: rufous fantail (*Rhipidura rufifrons*), bridled white-eye (*Zosterops conspicillatus*), and golden white-eye (*Cleptornis marchei*). Rainfall was positively related to vegetation greenness at all but the highest rainfall levels. Temporal variation in greenness affected the productivity of each bird species in unique ways. Predicted productivity of rufous fantail was highest when dry and wet season greenness values were high relative to site-specific 5-year seasonal mean values (i.e., relative greenness); while the white-eye species had highest predicted productivity when relative greenness contrasted between wet and dry seasons. Survival of rufous fantail and bridled white eye was positively related to relative dry-season greenness and negatively related to relative wet-season greenness. Bridled white-eye survival also showed evidence of a positive response to overall greenness. Our results highlight the potentially important role of rainfall regimes in affecting population dynamics of species on oceanic tropical islands. Understanding linkages between rainfall, vegetation, and animal population dynamics will be critical for developing effective conservation strategies in this and other regions where the seasonal timing, extent, and variability of rainfall is expected to change in the coming decades.

## Introduction

Remote oceanic islands are widely recognized as important reservoirs of regional and global biodiversity, and they are typified by endemic and range-restricted species with small global populations [1,2]. These island species face a variety of threats, including habitat loss and conversion, exotic invasive species, and climate change [3,4]; and they may be particularly vulnerable to extinction due to demographic and environmental stochasticity [5]. Understanding spatial and temporal variation of demographic rates could provide a critical tool for informing effective conservation efforts for island species. Yet, little is known about the population dynamics, trends, or demographic rates of most island species, or the environmental conditions that contribute to population changes [6,7].

Climatic variability may be particularly important in affecting the population dynamics of island species. Novel climatic conditions arising, in part, as a result of global climate change may threaten these populations in the future [8–10]. Climate change impacts on populations may range from direct effects, such as sea-level rise or creation of climatic conditions beyond physiological tolerance limits [11], to indirect effects on the spatial and temporal availability of resources and related intra- and inter-specific interactions [12]. On tropical islands, as across much of the mainland tropics, seasonal rainfall is the key climatic variable driving phenological patterns of plants [13,14]. Annual variability and trends in the timing or extent of rainfall can affect the availability of new leaves, flowers, and fruits available to herbivorous insect consumers [15,16], as well as to vertebrate consumers dependent on these plant and insect resources [17]. By linking demographic data on animal populations to time series of remote-sensed vegetation data [18], we can gain new insights into how animal consumers respond to spatial and temporal patterns of overall vegetation “greenness” (i.e., vegetation structure and productivity).

Here we report on a 5-year study of rainfall, vegetation greenness (enhanced vegetation index [EVI]) [19], and demographic rates (productivity, survival) of three endemic landbird taxa at six study sites on the island of Saipan in the Northern Mariana archipelago of Micronesia. Saipan is climatically similar to most other tropical Pacific oceanic islands, with temperatures that are relatively consistent throughout the year, and rainfall that is seasonally variable (most precipitation falling Jul-Nov). Rainfall can also be highly variable among years and is closely linked to the El Niño Southern Oscillation (ENSO) [20]. We expected that seasonal and annual variation in rainfall would result in concomitant changes in habitat phenology and landbird demography. We assessed three basic hypotheses about how demographic rates might vary as a function of vegetation condition: (i) demographic rates vary largely as a function of overall vegetation structure and plant productivity (average site-specific greenness across years and seasons); (ii) demographic rates depend on both structural and temporally varying components of greenness (year- and season-specific greenness at a site); and (iii) demographic rates depend largely on temporally varying components of greenness (i.e., plant productivity; greenness relative to site- and season [dry or wet]-specific annual mean). In general, we expected that aspects of greenness would be positively related to demographic rates. For example, a recent study of the Rota white-eye (*Zosterops rotensis*) on a nearby island suggested a positive relationship between bird density and leaf density [21]. Such positive relationships would suggest that vegetation density and productivity affects resource availability for birds and could be directly related to demographic parameters. However, we also expected that seasonal interactions might also play an important role in driving demographic rates. For example, positive effects of an unusually wet and green dry season on demographic rates (when resources might be most limiting) might reduce the magnitude of any wet-season greenness effects on demographic rates (i.e., a negative seasonal interaction).

## Materials and Methods

### Ethics statement

This research was conducted in compliance with the Guidelines to the Use of Wild Birds in Research (http://www.nmnh.si.edu/BIRDNET/guide/). The birds in this study were captured and banded under US federal bird banding permit 21731, which is overseen by the Northern Mariana Islands' Division of Fish and Wildlife (DFW). DFW secured all permissions to work on study areas.

### Focal bird species

The landbird fauna of the Northern Marianas includes 16 range-restricted (range < 50,000 km^2^) species (70% of the 23 total native landbird species) and 10 endemic species [22]; nine of these species are considered to be globally threatened [23]. We focus here on three taxa endemic to the Northern Mariana islands: rufous fantail (*Rhipidura rufifrons saipanensis*; subspecies occurs only on Saipan and Tinian); bridled white-eye (*Zosterops conspicillatus*; endemic to Tinian, Saipan and Aguiguan); and golden white-eye (*Cleptornis marchei*; endemic to Saipan and Aguiguan, prehistorically extirpated from Tinian) [24–27]. Rufous fantail and golden white-eye are of special conservation concern due to evidence of recent population declines [28].

### Study sites and field methods

**Fig 1:**
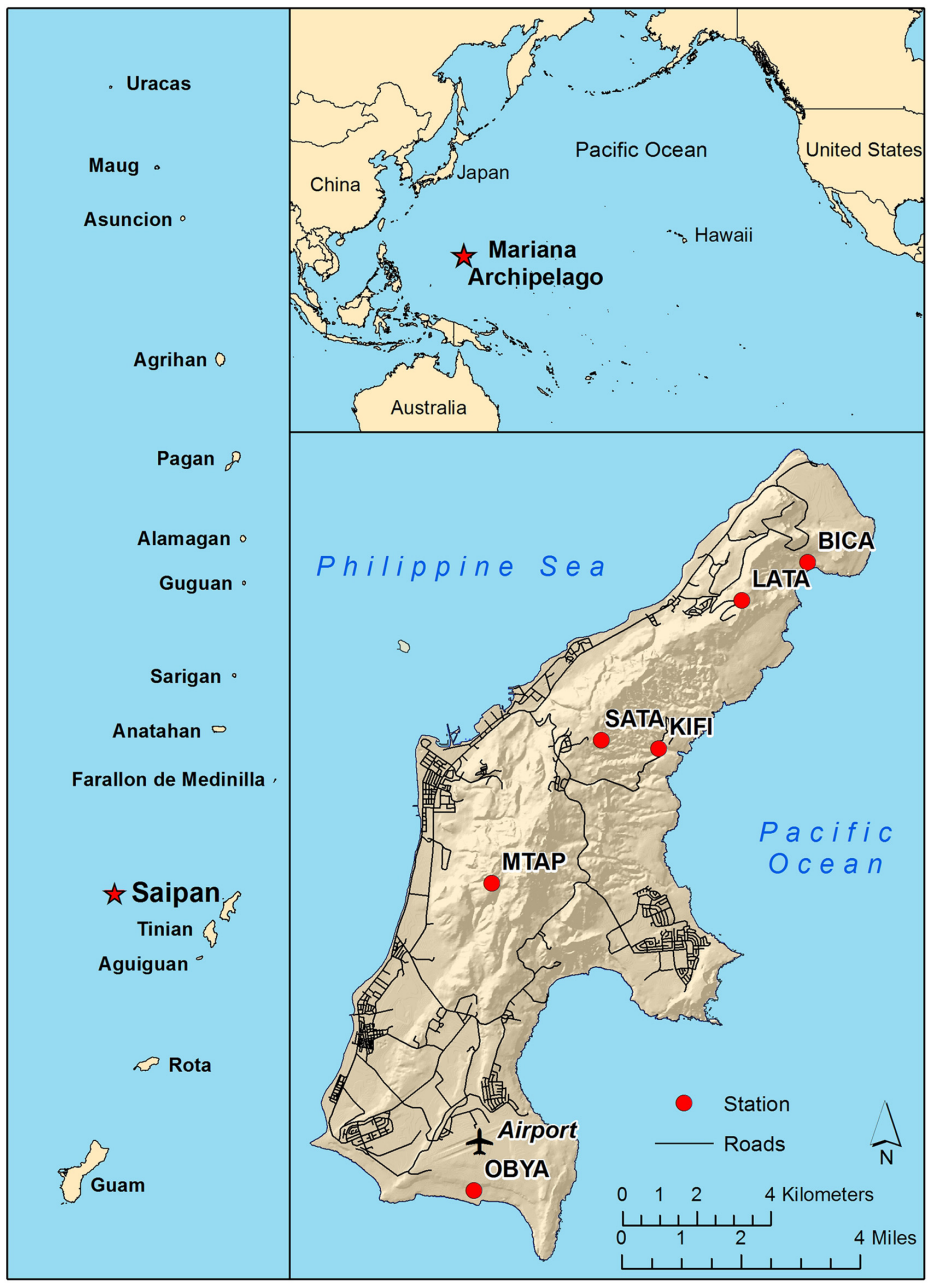
Locations of the Mariana Archipelago (top right), Saipan (left), and bird-banding stations operated as part of this study (bottom right). Station codes are defined in Table 1. The Saipan International Airport, where rainfall data were collected, is also shown.

We established six study sites in typical habitats utilized by landbirds on Saipan (Table 1; Fig 1). The island is composed of raised, terraced limestone formations culminating in a north-south ridgeline. Land cover types typical of the island include native limestone evergreen forest, mixed evergreen forest, tangan-tangan (*Leucaena leucocephala*) scrub, coastal scrub or strand vegetation, tropical savannahs, and swordgrass (*Miscanthus floridulus*) thickets. We selected study sites based on their composition of habitat representative of Saipan and nearby islands of Tinian and Rota (largely tangan-tangan and limestone forest) and their having a high likelihood of remaining intact for at least the five years of study reported here (no major disturbances occurred during the study period).

**Table 1:** Station names, codes (see Fig 1 for locations), major habitat types, geographic coordinates, elevations, and summary of annual effort.

					Effort (net-hours)^a^				
Station	Code	Habitat	Latitude, longitude	Elev. (m)	2008	2009	2010^b^	2011^b^	2012^b^
Bird Island Conservation Area	BICA	Tangan-tangan (*Leucaena leucocephala*) forest	15° 15' 45" N, 145° 48' 50" E	30	572.3	574.2	1407.7 (583.7)	1590.0 (567.3)	1066.7 (535.0)
Laderan Tangke	LATA	Lowland tropical rainforest and tangan-tangan forest	15° 15' 10" N, 145° 47' 54"E	207	520.5	522.2	1379.8 (584.0)	1579.0 (534.7)	1116.7 (537.3)
Sabana Talofofo	SATA	*Casuarina* savannah with swordgrass thicket	15° 13' 07" N, 145° 45' 44" E	161	414.7	429.0	1102.8 (463.5)	1351.0 (470.7)	957.3 (477.3)
Kingfisher	KIFI	Lowland tropical rainforest with riparian zone	15° 13' 02" N, 145° 46' 37" E	23	406.7	450.0	1033.3 (462.7)	1293.8 (450.5)	893.3 (449.3)
Mount Tapochau	MTAP	Submontane tropical rainforest	15° 11' 01" N, 145° 44' 04" E	274	421.7	454.0	1078.3 (462.7)	1295.3 (468.7)	847.3 (456.8)
Obyan	OBYA	Tangan-tangan forest	15°06'31"N, 145°43'49"E	1	561.2	543.5	1314.8 (539.0)	1594.3 (574.3)	1077.5 (518.5)

^a^ 1 net-hour = 1 12-m × 2.5-m mist net open for 1 hr.

^b^ Numbers in parentheses represent net-hours operated during the 10 sampling periods that were consistent among years (11 April-19 July).

At each study site, we established a bird mist-netting station consisting of eight to ten 12-m × 2.5-m, 30-mm mesh, 4-tier nylon mist nets erected at fixed net sites within an approximately 8-ha area. We operated each station on one day per 10-day period from 13 April-17 July 2008, 11 April-15 July 2009, 21 February-9 October 2010, 23 March-28 July 2011, and 1 April-13 July 2012 [29,30]. During July 2011 through March 2012 we operated stations for monthly pulses of three consecutive days, once per month [31]. In general, we operated nets for six morning hours per day of sampling (beginning at 05:30 AST). However, inclement weather (mostly high sun and wind exposure) and high capture rates at some sites resulted in slightly less and variable effort among stations and years. With few exceptions (< 3% of birds escaped from nets or were otherwise released unbanded), all birds captured in mist nets were identified to species, age (young = 'hatching year'; adult = 'after hatching year'), and sex [32] and banded with United States Geological Survey—Biological Resources Division numbered aluminum leg bands. Band numbers of all recaptures were carefully recorded. Although we recorded captures of 13 bird species, the large majority of captures (92%) were of the three species considered in our analyses. Of these, rufous fantail was the most commonly captured (4,083 captures, representing 51% of the total), followed by Bridled white-eye (1,444; 18% of total), and Golden white-eye (1,242 captures; 16% of total).

### Remote-sensed vegetation data and relationship to rainfall

We used monthly Enhanced Vegetation Index (EVI) data derived from the Moderate Resolution Imaging Spectroradiometer (MODIS) instrument of NASA's Terra satellite (http://terra.nasa.gov/) summarized at 1-km^2^ resolution (MODIS product MOD13A2) to describe patterns of vegetation greenness and to calculate covariates for productivity and capture-recapture analyses [19]. EVI is a composite metric of vegetation greenness; it incorporates structural and seasonal components of habitat quality, including primary productivity (leaf chlorophyll content), leaf area, canopy cover, and vegetation complexity [19,33,34]. EVI data are especially well-suited to studies of humid tropical forests (e.g., compared to the Normalized Difference Vegetation Index; NDVI) because it exhibits relatively low saturation at high values [35] and is relatively insensitive to clouds and smoke [36,37]. After removing cloud and aerosol contaminated pixels [38], we extracted interpolated monthly EVI values over the four 1-km^2^ pixels closest to station coordinates using the 'bilinear' option of the 'extract' function in the 'raster' R package [39].

We averaged the station-scale EVI values for each month between July 2007 and December 2012 and then averaged these values for each of the late dry season (Mar-May) and late wet season (Sep-Nov) for each year. We modeled these mean EVI values as a linear function of the 1-month lagged log-transformed mean monthly rainfall (in mm) during those seasons (i.e., we used rainfall data from Feb-Apr and Aug-Oct for the dry and wet seasons, respectively). Rainfall data were collected at the Saipan International Airport weather station and were provided by the NOAA National Climate Data Center (http://www.ncdc.noaa.gov/). We lagged rainfall data by 1-month to better match acquisition dates of MODIS data (beginning of the month) and the rainfall data (end-of-month sum).

### Avian productivity

Our analyses of avian productivity derive from basic methods described in Robinson et al.[40]. We assumed a binomial model for the proportion of young (hatching year) birds in the catch:

$$N_{st}^{Y}|(N_{st}^{Y}+N_{st}^{A})\sim \text{Bin}(N_{st}^{Y}+N_{st}^{A},p{\left[Y\right]}_{st}),$$

Where $N_{st}^{Y}$ is the number of young individuals captured at station *s* (where *s* = 1,…, 6 stations) in year *t* (where *t* = 1,…, 5 years; 2008–2012), $N_{st}^{A}$ is the number of adult (after-hatching-year) individuals captured at station *s* in year *t*, and *p*[*Y*]_*st*_ is the probability of an individual bird captured at station *s* in year *t* being a young bird. For summarizing $N_{st}^{Y}$ and $N_{st}^{A}$, we only included individuals captured during the ten 10-day sampling periods that were consistent among the three years (11 April-19 July). Sampling effort during this time was similar among years, ranging from a low of 2,897 net-hours in 2008 to a high of 3,095 net-hours in 2010. We excluded a small proportion (< 5% for all target species) of individuals for which we were unable to determine ages.

We considered a set of 16 logit-linear models to test hypotheses about effects of EVI on avian productivity. Our most general model was of the form:

$$\text{logit}(p{\left[Y\right]}_{st})=\beta_{0}+\beta_{1}+pr.ef_{st}+\sum_{i=2}^{4}\{\begin{array}{l} evi.mn_{s}\\ evi.w_{st-1}\\ evi.d_{st}\\ evi.w.dev_{st-1}+evi.d.dev_{st}+evi.w.dev_{st-1}:evi.d.dev_{st} \end{array},$$

where *β*_0_ was the intercept, *β*_1_was the effect of annual effort prior to the temporal window of the productivity analysis (*pr*.*ef*_*st*_), and the remaining *β*_*i*_ coefficients represented effects of one or more EVI covariates. We defined our prior-effort covariate, *pr*.*ef*_*st*_, as the log-transformed (+1) summed net-hours between the end of the previous year’s productivity time window and the start of the current year’s productivity time window. We considered *pr*.*ef*_*st*_ in models to correct for potential net avoidance that may have been induced by netting prior to the period over which we summed young and adult captures. We expected *p*[*Y*]_*st*_ would be positively related to *pr*.*ef*_*st*_ due to the likely greater exposure of adults to sampling (young would have likely been entering the population during the sampling period). We included 1–3 covariates in models that characterized spatial and temporal variation in EVI. To represent the hypothesis that productivity varied as a function of overall vegetation structure and productivity, we modeled productivity as a function *evi*.*mn*_*s*_, the average station-specific monthly EVI value across all five years of the study. To assess hypotheses that variation in productivity resulted from both structural and temporally varying aspects of greenness, we included station- and time-specific EVI covariates. The first of these, *evi*.*w*_*st*-1_, was the year- and station-specific monthly mean EVI during the late wet season (Sep-Nov) prior to the temporal window defined for productivity analyses. The second,*evi*.*d*_*st*_, was the year- and station-specific monthly mean EVI during the late dry season (Mar-May; time period overlapping the time window defined for the productivity analysis). We included only one of these two covariates in a given model, as they were highly correlated (*r* = 0.609, d.f. = 28; *P* < 0.001). Finally, to represent hypotheses that productivity varied largely as a function of annual variation in plant productivity during the wet and dry seasons, we considered covariates representing deviation of the *evi*.*w*_*st*-1_ and *evi*.*d*_*st*_ values from their station-specific wet and dry season averages across the five years of the study (e.g., for the wet season this would be $evi.w_{st-1}-\bar{evi.w_{s\cdot }}$). We denote these as *evi*.*w*.*dev*_*st*-1_ and *evi*.*d*.*dev*_*st*_. These deviation covariates were not strongly correlated (*r* = 0.082, d.f. = 28; *P* = 0.667); thus we considered additive and full interaction models with these two covariates. Covariates were standardized to mean zero and unit variance prior to analysis to facilitate estimation and interpretation.

We assessed support for the EVI covariate models based on Akaike's Information Criterion adjusted for small sample size (AIC_*c*_) and AIC_*c*_ model weights (*w*_*i*_, where here *i* = 1,…, 16 models;[41]). Models were implemented in the R statistical program[42], and we used functions in the R package MuMIn [43] for model selection.

### Avian survival probability

We used models developed for the joint analysis of mark-recapture and resighting-recovery data [44,45] to model capture-recapture data of adult (AHY) birds collected between April 11 and July 19 of each year and recaptures occurring between these months (our ‘resighting’ data in the context of the Barker model). The structure of the ‘Barker model’ allowed us to define sampling periods based on protocols that were consistent among years, while also exploiting recaptures occurring outside of these periods as supplemental data to inform estimation of survival and temporary emigration parameters. Despite their flexibility for handling capture-recapture data in the context of irregular annual sampling, Barker models have received little attention in a purely capture-recapture context [46].

The Barker model includes seven estimable parameters, including: (1) *S*, annual survival rate; (2) *p*, recapture probability of a marked individual during a regular sampling period (i.e. between Feb and May); (3) *F*, probability of site fidelity between years, (4) *F’*, probability of return for a temporary emigrant (i.e., probability of a marked individual not on the study area in time *t* returning to the study area in time *t +* 1); (5) *r*, the probability of recovering a dead marked individual between regular sampling periods (i.e., between May and Feb of the following year); (6) *R*, the probability of recapturing an individual between regular sampling periods given that the individual survives the interval between regular sampling periods; and (7) *R’*, the probability of recapturing an individual alive between regular sampling periods, given that the individual dies sometime between those regular sampling periods.

The Barker model can accommodate grouping structure and covariates to provide insights into factors that affect vital rates and detection parameters [44,47,48]. We focused most modeling efforts on the survival parameter, *S*. We interpret this parameter as apparent rather than true survival, as we set the fidelity parameter, *F*, to 1, and the return parameter, *F’*, to zero because all captures and recaptures were within the same study areas. We considered models for which survival was set as spatio-temporally constant (i.e., S[.] models) as well as models that allowed *S* to vary as a function of various EVI covariates analogous to those defined for the productivity models. EVI effects included *evi*.*d*_*st*_ (mean dry-season [Mar-May] EVI at station *s* and year *t*) *evi*.*w*_*st*_ (mean wet-season [Sep-Nov] EVI at station *s* and year *t*), *evi*.*d*.*dev*_*st*_ (deviation of dry-season EVI at station *s* year *t* from the 5-yr [2008–2012] mean dry-season EVI at station *s*), *evi*.*d*.*dev*_*st*_(deviation of wet-season EVI at station *s* and year *t* from the 5-yr [2008–2012] mean wet-season EVI at station *s*), and *evi*.*mn*_*s*_, the mean EVI value across the 5-yrs of the study. We considered all combinations of models for *S* including no space-time effects, single EVI covariate effect models, and additive and full interaction models including the *evi*.*d*.*dev*_*st*_ and *evi*.*w*.*dev*_*st*_ variables.

We modeled the remaining model parameters of the Barker model as follows. First, we set *r* to zero, because no individuals were ever recovered dead, and no effort was expended in searching for dead birds. A very small number of individuals (16) was either found dead in mist nets, or died prior to release, presumably as a result of injury due to mist-netting. We excluded these individuals from our analysis. We modeled *p* as either time-constant or as a function of year. We modeled R and R’ as constant across space and time, with the exception that we fixed these to zero for the interval between 2008 and 2009 (no netting effort between periods) and for the interval after 2012 (again, no effort after July in 2012).

Models were run in program MARK [49] using the R [42] package RMark [50]. We assessed goodness-of-fit for each species using the median $\hat{c}$ procedure in program MARK using simulated data sets based on the most parameterized model. Estimates did not suggest substantial overdispersion ($\hat{c}$ ranging from 1.02 for bridled white-eye to 1.16 for rufous fantail) and adjustments to $\hat{c}$ did not affect model selection. Thus, we compared models using AIC corrected for small sample size, AIC_*c*_, and assessed model support using AIC_*c*_ model weights (*w*_*i*_, where *i* = 1,…, 16 models; [41].

## Results

### Remote-sensed vegetation data and relationship to rainfall

**Fig 2:**
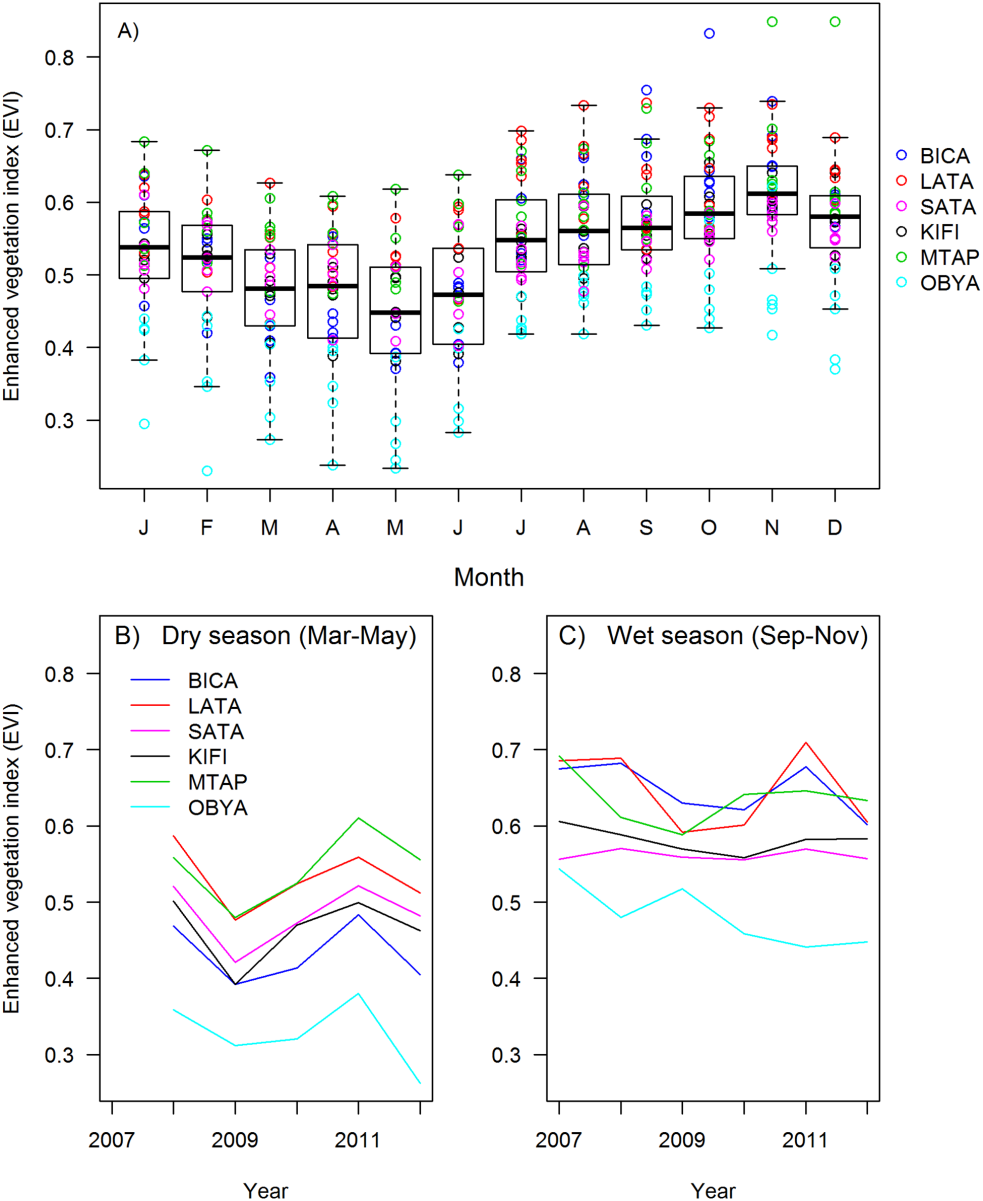
Station-scale MODIS-derived enhanced vegetation index (EVI) values. EVI values represent interpolated monthly EVI values over the four 1-km^2^ pixels closest to station coordinates. EVI values are plotted (A) by month (individual points represent year-specific values for each station; boxplots delineate quartiles with whiskers bounding the 95^th^ percentile) and (B-C) by year during the late dry (B: Mar-May) and wet (C: Sep-Nov) seasons.

EVI values varied among stations, between wet and dry seasons, and among years (Fig 2). Mean monthly EVI values were lowest at the most southerly and lowest elevation station, OBYA (0.41); and highest at the high-elevation sites, MTAP (0.59) and LATA (0.60). EVI values were lowest late in the dry season (Mar-May) and highest during the late wet season (Sep-Nov; Fig 2A). The pattern of annual variation in EVI during the dry season was similar among stations, with peaks occurring in 2008 and 2011 and lowest values in 2009 (Fig 2B). Patterns in annual variation in wet-season EVI were less clear, although all stations except OBYA had relatively high EVI in 2011 (Fig 2C).

**Fig 3:**
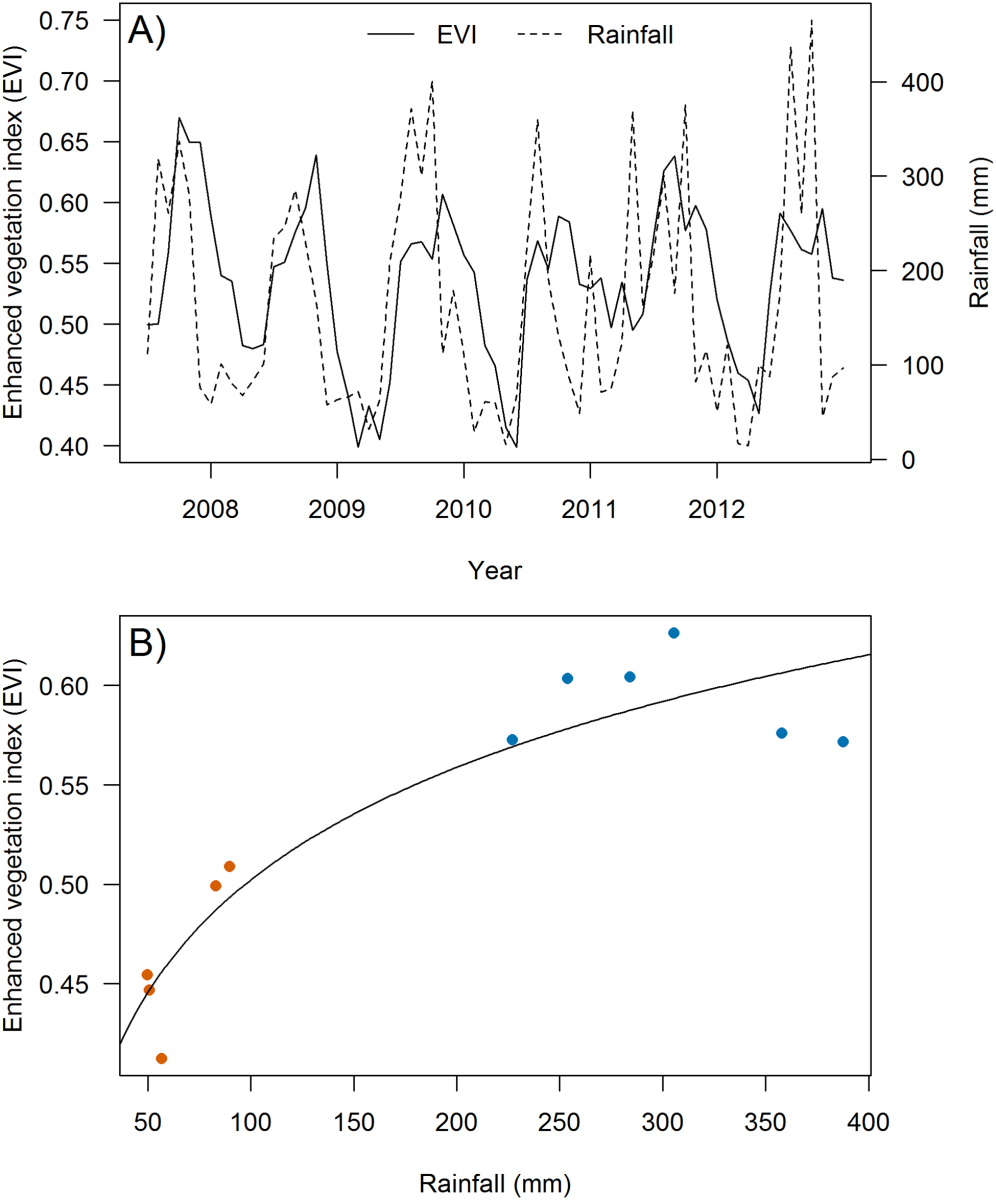
EVI-rainfall patterns. (A) Time series showing seasonal and annual variation in monthly rainfall recorded at the Saipan International Airport and average monthly EVI values at the six mist-netting stations on Saipan during Jul 2007-Dec 2012 and (B) Relationship between monthly mean EVI and rainfall. Curve shows log-linear model fit; red dots represent dry-season means for the six mist-netting stations and blue dots represent wet-season means.

Monthly variation in EVI was positively related to rainfall, although there was some indication that EVI may have been depressed at the highest rainfall values (> ~ 350 mm; Fig 3). Rainfall and EVI were strongly seasonal with distinct wet and dry seasons (Fig 3A). The rainfall covariate (on log-scale) in our regression model was significant ($\hat{\beta }$ = 0.082; SE = 0.011; *P* < 0.0001; Fig 3B).

### Avian productivity

**Fig 4:**
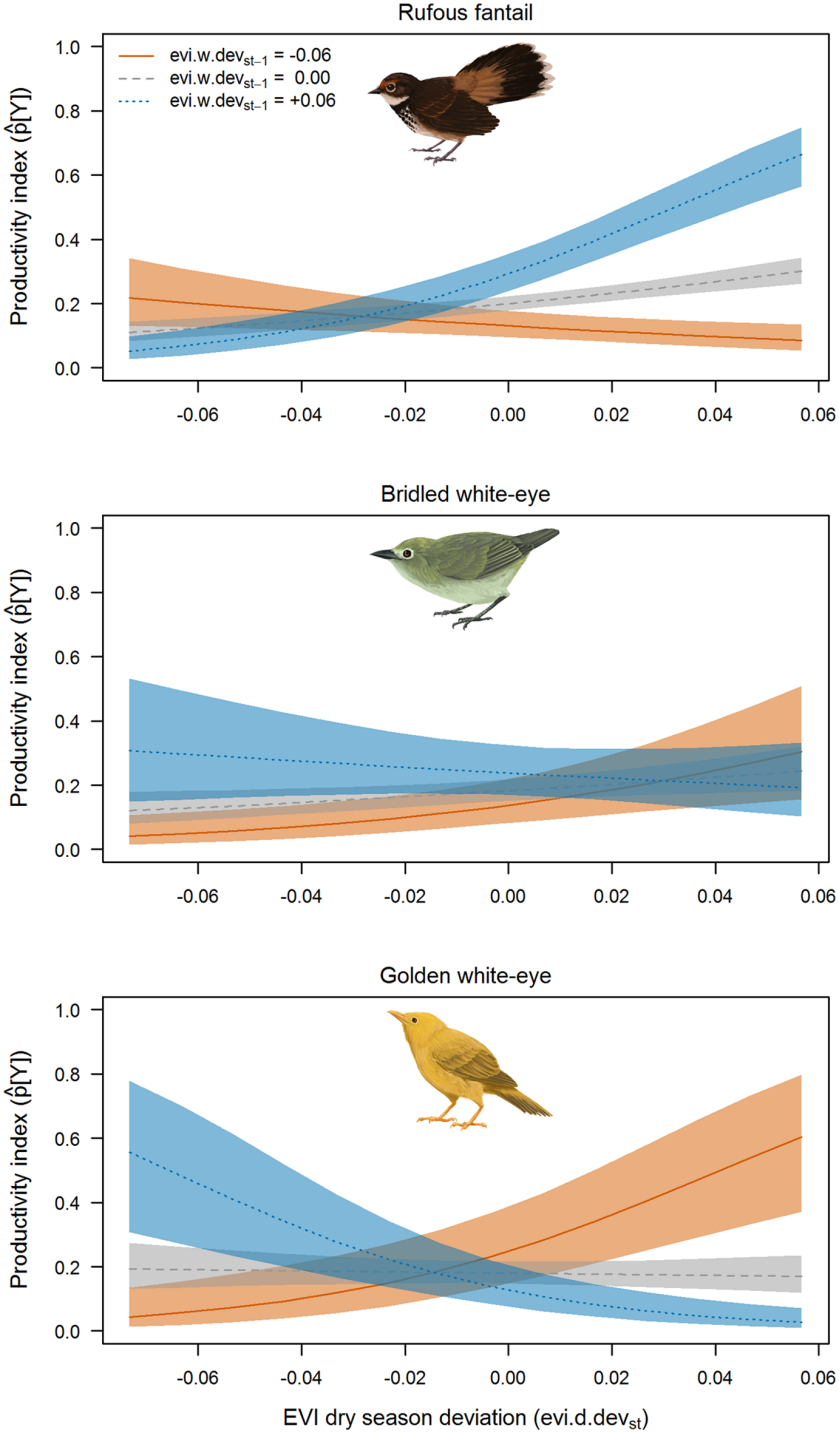
EVI-avian productivity relationships. Predicted productivity (probability of capturing a hatching-year bird; *p*[*Y*]_*st*_ ± 95% confidence intervals) in relation to deviation of the enhanced vegetation index (EVI) from 5-year mean values during the late dry season (Mar-May; *evi*.*d*.*dev*_*st*_) at three levels of late wet season EVI deviation values (from previous Sep-Nov; *evi*.*w*.*dev*_*st-1*_). Predictions are based on top-performing (lowest AIC_*c*_) models for the three target species using capture data collected during the 10 periods (11 April-19 July) sampled in each of the five years (2008–2012).

We found strong support for effects of deviation of wet and dry season EVI values from their station-specific seasonal means (*evi*.*w*.*dev*_*st*-1_ and *evi*.*d*.*dev*_*st*_), i.e., relative greenness, on avian productivity for all three focal species (Table 2; Fig 4). The best model for all species was the full interaction model (*evi*.*w*.*dev*_*st*-1_+*evi*.*d*.*dev*_*st*_+*evi*.*w*.*dev*_*st*-1_:*evi*.*d*.*dev*_*st*_). We found nearly all support for this model for rufous fantail (*w*_*i*_, = 1.00) and golden white-eye (*w*_*i*_, = 0.99). We found slightly less support for this model for bridled white-eye (*w*_*i*_, = 0.70; all other models with *ΔAIC*_*c*_ > 3). Rufous fantail and golden white-eye showed the strongest responses, and the effects of temporal variation in EVI on productivity differed markedly for the two species (Fig 4). For rufous fantail, predicted productivity was highest when both wet and dry season EVI were relatively high. However, in years following low-EVI wet seasons, predicted productivity was relatively low regardless of how high EVI was during the dry season. In contrast, golden white-eye productivity was highest when EVI deviation contrasted between wet and dry seasons. Bridled white-eye showed a response that was similar to, albeit weaker than, the response exhibited by golden white-eye. As expected, we found sampling effort in the interval prior to the productivity sampling window (*pr*.*ef*_*st*_) to positively affect the productivity index (Table 2).

**Table 2:** Sample sizes (age-specific year-unique captures between Apr 11 and Jul 19 of 2008–2012) and standardized regression coefficients (95% confidence intervals) for effects included in top models examining hypotheses relating the enhanced vegetation index (EVI) to avian productivity.

Species	*N*^*Y*^	*N*^*A*^	*pr*.*ef*_*st*_	*evi*.*w*.*dev*_*st-1*_	*evi*.*d*.*dev*_*st*_	*evi*.*w*.*dev*_*st-1*_:*evi*.*d*.*dev*_*st*_
Rufous fantail	423	1341	0.73 (0.56, 0.90)	0.28 (0.12, 0.44)	0.46 (0.34, 0.60)	0.41 (0.27, 0.54)
Bridled white-eye	137	545	0.64 (0.39, 0.91)	0.21 (-0.04, 0.47)	0.20 (-0.01, 0.41)	-0.25 (-0.47, -0.04)
Golden white-eye	141	541	0.51 (0.21, 0.80)	-0.27 (-0.56, 0.02)	-0.16 (-0.38, 0.06)	-0.57 (-0.81, -0.33)

*N*^Y^ = hatching-year birds and *N*^*A*^ = after-hatching-year birds.

### Capture-recapture models

#### Avian *s*urvival probability

**Fig 5:**
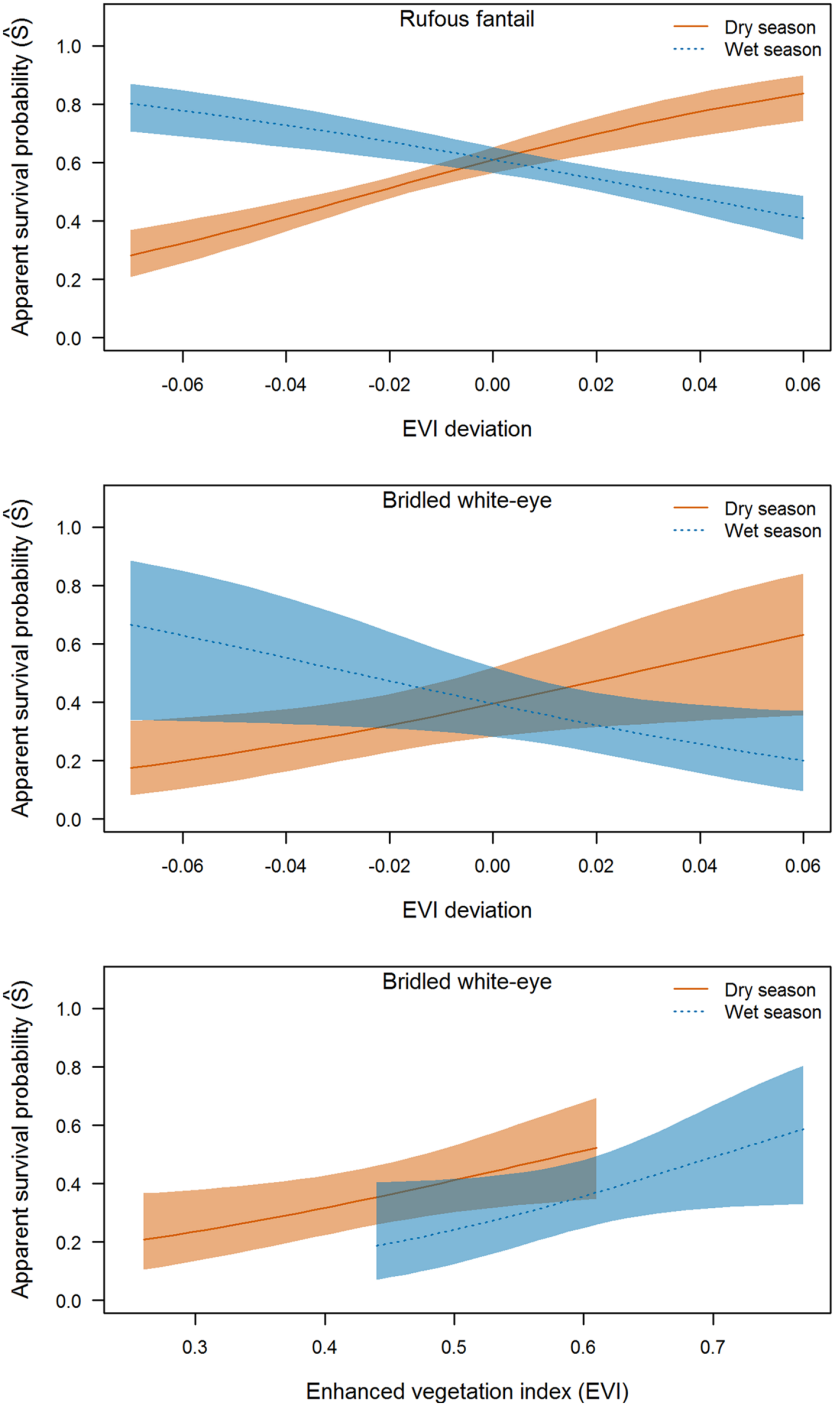
EVI-avian survival relationships. Predicted adult apparent survival probability ($\hat{S}$) in relation to: (top and middle panels) deviation of the enhanced vegetation index (EVI) from 5-year mean values during the late dry (Mar-May; *evi*.*d*.*dev*_*st*_) and late wet (Sep-Nov; *evi*.*w*.*dev*_*st*_) seasons from best (lowest AIC_*c*_) model (*evi*.*d*.*dev*_*st*_+*evi*.*w*.*dev*_*st*_); and (bottom panel) annual mean EVI during dry (*evi*.*d*_*st*_) and wet (*evi*.*w*_*st*_) seasons for bridled white-eye based on models with similar support to the top model.

We found support for EVI effects on adult apparent survival for rufous fantail and bridled white-eye (Table 3). For rufous fantail, the best (lowest *AIC*_*c*_) model for survival included additive *evi*.*w*.*dev*_*st*_ and *evi*.*d*.*dev*_*st*_ effects. Although the model including an interaction term for these two effects was also within 2 *AIC*_*c*_ points of the best model, the deviance explained by the model was nearly identical to the simpler model and the regression coefficient for this effect was estimated with low precision ($\hat{\beta }$ = 19.7; *SE* = 66.4; 95% CI = -110.5–149.8). The top model for bridled white-eye was also the *evi*.*w*.*dev*_*st*_ + *evi*.*d*.*dev*_*st*_ model, although model selection uncertainty was greater for this species (Table 3). For both species, predicted survival estimates based on the top model suggested that survival was positively related to dry season EVI deviation and negatively related to wet season EVI deviation (Fig 5). For bridled white-eye, models including *evi*.*d*_*st*_ and *evi*.*w*_*st*_ effects received similar support to the top model and the regression coefficients for these effects were positive and similar in magnitude, suggesting overall positive effects of greenness ($\hat{\beta }$ = 4.10; *SE* = 1.80; 95% CI = 0.58–7.62 for the *evi*.*d*_*st*_ model and $\hat{\beta }$ = 5.54; *SE* = 2.91; 95% CI = -0.18–11.25 for the *evi*.*w*_*st*_ model; Fig 5). There was considerable model uncertainty for the survival models for golden white-eye (Table 3), and little support for EVI effects on survival for this species (no EVI regression coefficients significant).

**Table 3:** Model selection results from Barker capture-recapture models applied to data on three bird species from six mist-netting stations on Saipan, 2008–2012. Only models within 2 *AIC*_*c*_ points of the best model are shown.

		No. recaptures		Model			
Species	No. individuals	Primary	Interval	*S*	*p*	No. parameters	*AIC*_*c*_ weight (*w*_*i*_)
Rufous fantail	1088	242	680	*evi*.*w*.*dev*_*st*_ *+ evi*.*d*.*dev*_*st*_	*year*_*t*_	9	0.72
				*evi*.*w*.*dev*_*st*_ *+ evi*.*d*.*dev*_*st*_ *+ evi*.*w*.*dev*_*st*_: *evi*.*d*.*dev*_*st*_	*year*_*t*_	10	0.28
Bridled white-eye	519	25	62	*evi*.*w*.*dev*_*st*_ *+ evi*.*d*.*dev*_*st*_	·	6	0.23
				*evi*.*d*_*st*_	·	5	0.20
				*evi*.*w*_*st*_			0.15
				*evi*.*mn*_*s*_	·	5	0.10
Golden white-eye	464	77	128	·	·	4	0.24
				*evi*.*w*_*st*_			0.16
				*evi*.*w*.*dev*_*st*_	·	5	0.10
				*evi*.*w*.*dev*_*st*_ *+ evi*.*d*.*dev*_*st*_	·	6	0.10
				*evi*.*mn*_*s*_	·	5	0.10
				*evi*.*d*.*dev*_*st*_	·	5	0.09
				*evi*.*d*_*st*_	·	5	0.09

#### Recapture probability

Top models for recapture probability for rufous fantail included year effects, and recapture probability estimates declined across the years of the study ranging from $\hat{p}$ = 0.402 (95% CI: 0.321–0.489) in the first recapture year of 2009 to $\hat{p}$ = 0.159 (95% CI: 0.120–0.208) in 2012. Model selection results suggested time-constant recapture probability for both white eye species for which recapture probability estimates were similarly low: $\hat{p}$ = 0.113 (95% CI: 0.007–0.186) for bridled white-eye and $\hat{p}$ = 0.184 (95% CI: 0.139–0.240) for golden white-eye.

## Discussion

Our results indicated strong links between rainfall, vegetation greenness, and the demographic rates of three endemic island landbirds. Rainfall was positively associated with vegetation greenness in both dry and wet seasons, although greenness was lower than expected at the highest rainfall levels recorded. This observation is consistent with other studies of tropical forests that show steep increases in plant productivity up until about 2500 mm of rainfall/year, beyond which plant productivity flattens or diminishes [33,51].

Of the three hypotheses considered for explaining links between demographic rates and vegetation greenness, nearly all support was for the notion that temporal variation in greenness was the principal driver of demographic rates. Although we predicted that demographic rates would be associated with higher EVI values, relationships were more nuanced and species-specific. Productivity of rufous fantail appeared to benefit from higher than normal vegetation greenness in the dry season; however, because of seasonal interactions, this positive response to dry season greenness may be limited to years following relatively green wet seasons. In contrast, golden white-eye, and to a lesser extent, bridled white-eye, had highest predicted productivity in years where relative greenness contrasted between wet and dry seasons. Although timing of reproduction appears to be flexible in all three focal species [52,53], a breeding peak seems to occur during the late-wet/early-dry season. It is possible that heavy rainfall events at that time could negatively affect nest success [54] and be reflected in years with similar relative greenness between wet and dry seasons. Such a mechanism may have contributed to the observed predicted productivity pattern in white-eyes; however, it is not clear why such an affect would not also have been evident for rufous fantails.

Species differences in responses of productivity to vegetation may have reflected differences in foraging niches and diets [55]. For example, rufous fantails tend to forage on aerial insects and glean from live leaves [52,53], and as such may benefit from wetter conditions that favor many herbivorous prey species [56]. Golden white-eyes, on the other hand, tend to forage more at dead leaves [57] where prey species such as detritivorous and scavenging insects may be favored under conditions in which especially dry seasons follow especially wet seasons [58]. Such conditions may be common in this region where dry years tend to follow wet El Niño years [20]. In addition, both white-eye species include fruit in their diet, while rufous fantails are strictly insectivorous [57]. Persistently low or high greenness values might disrupt flowering and fruit set of tree species utilized by white-eyes [13], reducing food resources for these species relative to rufous fantails. Thus, overall differences in productivity responses among species could have resulted from differential effects of rainfall and greenness on resource availability.

In contrast to productivity, we did not find evidence of seasonal interactions with respect to adult apparent survival. It is possible that cumulative greenness effects on survival occur across time scales longer than considered here (e.g., several years of low food availability). However, additional years of monitoring and analyses will be needed to assess this possibility. Adult apparent survival rates for rufous fantail and bridled white-eye were positively related to relative site- and year-specific dry-season greenness and negatively associated with relative wet-season greenness. For bridled white-eye, we also found evidence for an overall positive effect of greenness on survival. The positive relationships between dry-season greenness and survival are consistent with our hypothesis that greenness would generally correspond to increases in potential food resources and demographic rates. Drought conditions have been shown to negatively affect survival of migrant and resident birds in other systems with ENSO-driven rainfall patterns [59,60]. The mechanism for the negative relationship between relative wet-season greenness and survival is less clear. This relationship could reflect a situation where relatively consistent moderate conditions (relatively high dry-season greenness and low wet season greenness) favor survival, while more extreme or variable conditions favor productivity. It is also possible that extreme rainfall events, which may be more likely in high rainfall years, could negatively affect survival. Research currently underway is aimed at understanding seasonal components of demographic rates and how they relate to temporal variation in habitat quality.

It should be noted that because of competing research objectives and annual funding variation, we sampled landbirds across different temporal windows in most years. Nevertheless, we feel that our analytical approaches effectively accounted for effects of any potential sampling biases on inferences and made best use of all available data. For example, by allowing a particular year’s productivity index to depend on prior effort in that year, we were able to control for potential age-specific differences in exposure to netting and responses to capture. Our implementation of Barker capture-recapture models based on a fixed annual sampling period with supplemental recapture data between periods made efficient use of the extra data provided in years with extended sampling [46]. In addition, by allowing recapture probabilities to vary as a function of year, we accounted for any potential influence of net avoidance related to extended sampling in some years in affecting recapture rates [61]. Annual declines in recapture probability for rufous fantail across years suggested that net avoidance was an important issue affecting recapture probabilities of at least that species.

Given a dearth of data on Micronesian landbird populations [21,28,52,55,57,62–65], our study represents an important advance in informing the conservation of these species. The need for understanding the environmental drivers of demographic rates and population dynamics of these species is pressing given their high conservation priority [1,2], the many threats to the persistence of their populations (e.g., habitat loss, introduced species [28,62]), and the inherent vulnerability of their populations to environmental and demographic stochasticity. Understanding the role of climate variation in affecting plant and animal populations on islands of the tropical Pacific region, in particular, should be of high priority, because climatic conditions are projected to become warmer and wetter, and potentially more variable, in the coming decades [10,66–68]. On Saipan and the rest of the Mariana Islands, both dry and wet seasons are expected to receive more rainfall in the future [68]. Although such conditions may alleviate potential drought conditions in some years, they could also have potentially negative consequences for species like golden white-eye, which may have higher reproductive output in years with contrasting wet and dry seasons. We suggest that identifying population responses such as these to seasonal and annual climate variation should be an integral component of efforts to model viability of island species under varying climate change scenarios.
